# Durvalumab-Associated Myocarditis Initially Presenting With Sinus Bradycardia Progressing Into Complete Heart Block

**DOI:** 10.7759/cureus.40171

**Published:** 2023-06-09

**Authors:** Suhwoo Bae, Michael Vaysblat, Jason Ng, Nicholas Beccarino, John Makaryus, Kumar Sarkar

**Affiliations:** 1 Internal Medicine, Donald and Barbara Zucker School of Medicine at Hofstra/Northwell, Manhasset, USA; 2 Cardiology, Donald and Barbara Zucker School of Medicine at Hofstra/Northwell, Manhasset, USA

**Keywords:** cardiac conduction disorder, cardiac oncology, cardiac electrophysiology, ici, complete heart block

## Abstract

Durvalumab is a monoclonal antibody that blocks programmed cell death ligand-1 (PD-L1). It has been recently approved for the treatment of advanced urothelial and non-small cell lung cancer (NSCLC) with a more favorable side effect profile compared to traditional chemotherapy agents. We present a case of durvalumab-induced myocarditis complicated by complete heart block (CHB). A 71-year-old male with a history of atrial flutter status post ablation, type 2 diabetes mellitus, hypertension, and non-small cell lung carcinoma (NSCLC) recently started on durvalumab, presented with new sinus bradycardia on electrocardiogram (EKG). His initial labs were notable for a troponin T of 207 ng/L (normal range ≤50). Transthoracic echo (TTE) and computed tomography angiography (CTA) of the coronaries were unremarkable. The hospital course was complicated by CHB on telemetry for 15 minutes. Given hemodynamic instability, cardiac magnetic resonance imaging (MRI) could not be obtained. The patient received transvenous pacing. Electrophysiology and cardiology-oncology were consulted to evaluate for pacemaker implantation as well as management for durvalumab-induced myocarditis. Methylprednisolone 1000 mg intravenous (IV) was started with an improvement in troponin levels but without improvement in CHB. His course was further complicated by polymorphic ventricular tachycardia prompting the placement of a permanent dual-chamber pacemaker. The patient was discharged on a prednisone taper, and durvalumab was discontinued.

A diagnosis of durvalumab-induced myocarditis was made based on elevated troponin levels, with the exclusion of coronary artery disease with CTA of the coronaries. The persistence of conduction abnormalities despite treatment with steroids leads to the placement of a permanent pacemaker. Durvalumab falls under the category of immune checkpoint inhibitor (ICI) therapy which are novel agents that have more favorable side effect profiles compared to traditional chemotherapeutic agents. A review of the literature shows myocarditis with arrhythmias as a potentially rare side effect of ICI therapy. Corticosteroid therapy seems to be promising as a potential therapy.

## Introduction

Immune checkpoint inhibitors are novel immunotherapy agents that block checkpoint proteins such as programmed cell death protein 1 (PD-1), programmed cell death ligand-1 (PD-L1), or cytotoxic T-lymphocyte-associated protein 4 (CTLA-4) via monoclonal antibodies. Checkpoint proteins act as regulators of the immune system and are involved in aiding tumor cells in avoiding recognition by an immune response. Durvalumab is an FDA-approved immunotherapy that specifically binds to PD-L1 and was originally designated by the FDA for breakthrough therapy of metastatic urothelial bladder cancer whose tumor cells expressed PD-L1. It was then later approved for unresectable stage III non-small cell lung carcinoma (NSCLC) showing a longer median overall survival (16.3 months) compared to chemotherapy (12.9 months) in patients with PD-L1 ≥25% [1].

Durvalumab seems to have a tolerable safety profile with major adverse events being pruritus, fatigue, decreased appetite, diarrhea, transaminitis, nausea, and rash based on a recent meta-analysis [2]. While rare, immune checkpoint inhibitor-associated cardiotoxic effects seem to be associated with higher mortality with most cardiotoxic effects appearing to be inflammatory in nature [3]. Here, we present a case of a male receiving durvalumab for NSCLC with myocarditis presenting as sinus bradycardia with progression to complete heart block (CHB) and complicated by polymorphic ventricular tachycardia treated with systemic pulse-dose steroids and pacemaker implantation.

This article was previously presented as a meeting abstract at the 2023 JACC Annual Scientific Meeting on March 4, 2023.

## Case presentation

**Figure 1:**
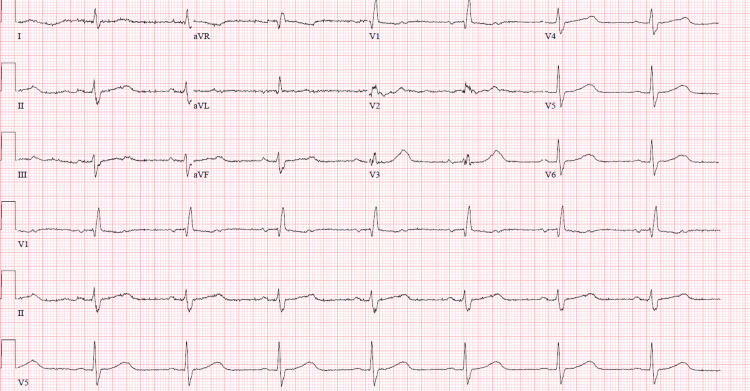
Electrocardiogram on admission demonstrating sinus bradycardia with bifascicular block

A 71-year-old male with a past medical history of atrial flutter status post ablation, type 2 diabetes mellitus, hypertension, chronic obstructive pulmonary disease, and stage T2N3M0 NSCLC receiving durvalumab 1500 mg IV every four weeks for NSCLC presented from his cancer center with bradycardia. He originally presented to his cancer center to receive his fourth infusion of durvalumab but was sent to the emergency department (ED) after vital signs showed bradycardia to the 40s. He had one week of dyspnea, intermittent chest pain, and dizziness without syncope. In the ED, vital signs were remarkable for bradycardia to the 40s compared to a baseline of 70-80, and examination showed diffuse bilateral rhonchi to auscultation. His initial troponin T, high-sensitivity assay was 207 mg/mL (normal range ≤50) with repeats elevated between 150-200 mg/ml. The pro-brain natriuretic peptide was 973 pg/mL. His EKG showed 1st-degree atrioventricular (AV) nodal delay with right bundle branch block and left anterior fascicular block, unchanged from prior (Figure 1).

**Figure 2:**
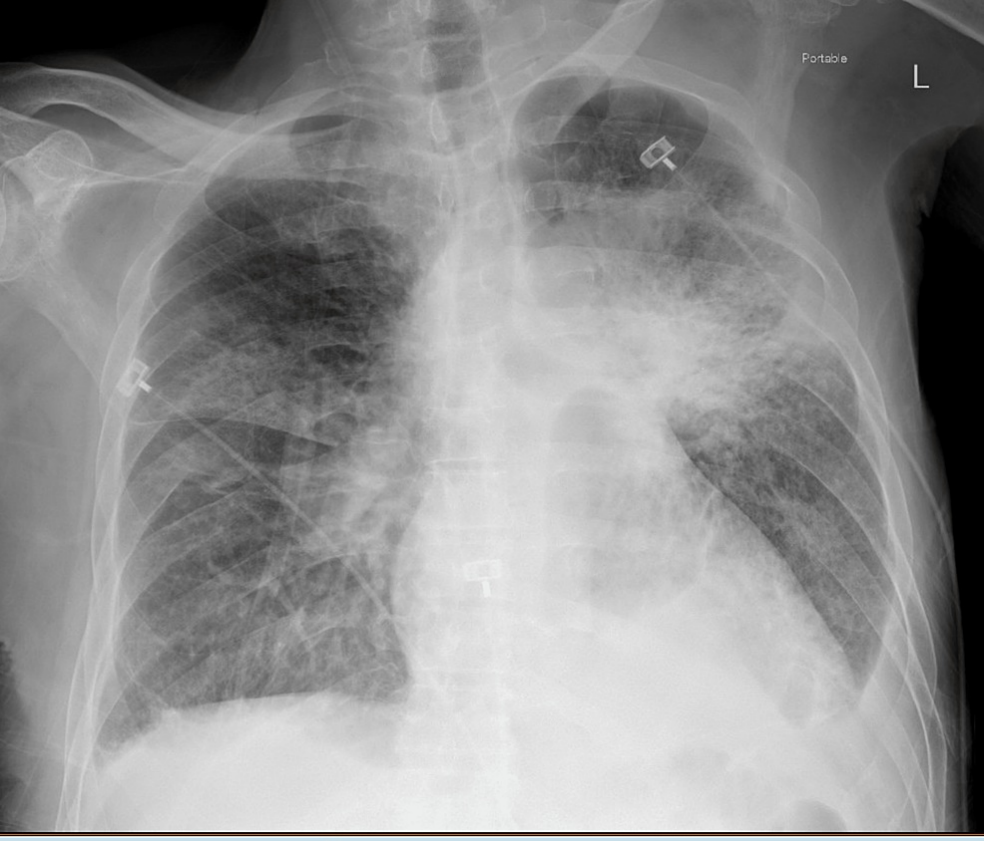
Chest radiography image on admission The image is showing a trace right pleural effusion and a small left pleural effusion, bilateral hazy opacities with normal heart size.

Chest radiography showed trace right and small left pleural effusions with diffuse bilateral hazy opacities, also unchanged from prior (Figure 2).

**Figure 3:**
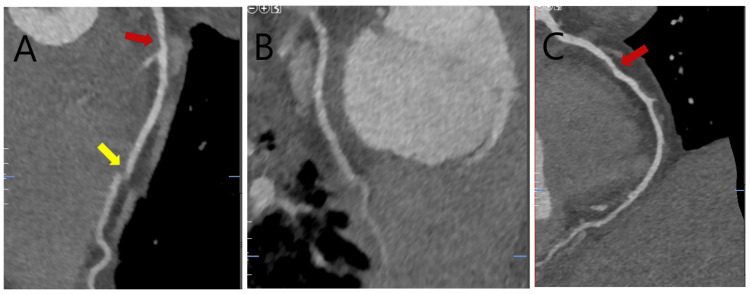
Curved multiplanar reformatted multidetectory coronary CT angiography images LAD: Left anterior descending artery; RCA: Right coronary artery Assessment of some segments of the coronary arteries was limited by motion and stitch artifacts. The LAD (image A) contained a region of non-calcified plaque with mild (less than 50%) luminal stenosis in the mid-segment (red arrow). A segment of the distal LAD was not well evaluated due to stitch artifact (yellow arrow). The evaluable segments of the left circumflex coronary artery (image B) revealed no obstructive coronary artery disease. The right coronary artery (image C) contained non-calcified plaque in the mid-segment with mild (less than 50%) luminal stenosis (red arrow).

CTA of the coronaries showed a total coronary artery calcium score of 0 and a non-calcified plaque within the mid-left anterior descending artery (LAD) and the right coronary artery (RCA) which were estimated to be less than 50% stenosis (Figure 3).

Transthoracic echo (TTE) was performed showing mitral annular calcification with mild mitral regurgitation but otherwise was unremarkable with an estimated left ventricular ejection fraction of 66%.

**Figure 4:**
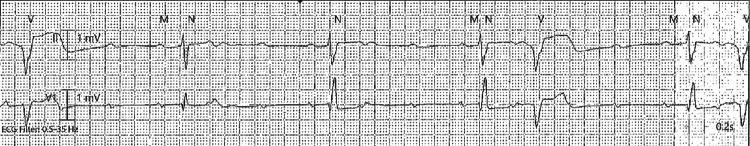
Telemetry strip showing complete heart block

On day two of hospitalization, the patient had a 15-minute episode of CHB on telemetry (Figure 4) that resolved spontaneously, requiring transfer to the coronary care unit for monitoring.

Repeat TTE was unchanged (Video 1).

**Video 1:** Repeat TTE day two of hospitalization TTE: Transthoracic echo No evidence of structural or functional abnormalities.

**Figure 5:**
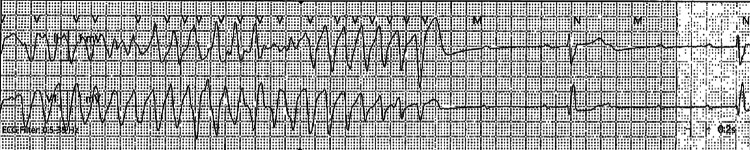
Telemetry strip showing polymorphic ventricular tachycardia

Cardiac MRI was deferred as the patient developed polymorphic ventricular tachycardia (Figure 5) requiring an emergent transvenous pacer with overdrive pacing.

The patient was started on methylprednisolone 1000 mg IV daily with guidance from cardio-oncology for presumed durvalumab-associated myocarditis leading to the resolution of troponinemia. His heart rhythm intermittently converted from CHB to sinus bradycardia for five days after corticosteroid initiation. He underwent successful placement of a permanent dual-chamber permanent pacemaker (PPM) and was discharged on a three-week prednisone taper.

The patient was seen by his primary oncologist and cardiology oncologist. The decision was made to discontinue his durvalumab treatment with the resolution of initial symptoms and normal cardiac markers two weeks post-admission.

## Discussion

We present a case of CHB caused by suspected ICI-associated myocarditis after three months of durvalumab therapy, successfully treated with corticosteroids and PPM implantation. ICIs are valuable immunotherapy agents in treating malignancies but have been found to be associated with cardiotoxicity including left ventricular dysfunction, arrhythmia, and conduction abnormalities.

A 2017 descriptive observational analysis observed that ICI-associated cardiotoxicity manifested mostly as left ventricular systolic dysfunction, atrial fibrillation, and ventricular arrhythmia at 79%, 30%, and 27% respectively. This was primarily seen in ipilimumab, nivolumab, pembrolizumab, and atezolizumab. Conduction abnormalities were rare at 17%. Of note, this study only included 30 patients likely because ICI agents were novel at that time and did not differentiate between different conduction disorders [4].

A 2021 retrospective analysis using data from the Food and Drug Administration Adverse Event Reporting System attempted to analyze the association between arrhythmias and ICIs. It included 1945 reports of suspected ICI-related arrhythmic events. This study reported atrial fibrillation as the most common arrhythmia with 576 reports (29.6%) and conduction disorders, which were defined as atrioventricular blocks and bundle branch blocks, were reported in 200 cases (10.3%). Except for cardiac arrest, conduction disease had the second highest fatality rate at 23.50% of cases resulting in death although, once again, the fatality rates did not differentiate between different conduction disturbances such as CHB, Mobitz II second-degree AV nodal block, and bundle branch blocks. This study also reported that lung cancer patients were the most likely to experience an adverse arrhythmic event (32.08%) [5] which was also confirmed by a 2022 systematic review including more than 40 clinical trials and case reports of 14 different cancers [6]. Among all patients on ICI monotherapy with an adverse arrhythmic event, durvalumab was reported to have the highest fatality rate at 33.93% [5]. Combination therapy is also reported to be associated with higher rates of myocarditis [7].

Other case reports have demonstrated ICI-associated myocarditis that has successfully been treated with corticosteroids with the resolution of arrhythmias [8,9]. Our case differs in that the patient had persistence of CHB even with the resolution of troponinemia after receiving corticosteroids. A 2020 international multicenter retrospective study examined the relationship between corticosteroids and major adverse cardiac events (MACE) defined by the study as cardiovascular death, cardiac arrest, cardiogenic shock, and hemodynamically significant complete heart block requiring PPM. There was a statistically significant relationship between both the dose of corticosteroids as well as the timing of the first dose received from admission to MACE. Both high-dose steroids (501-1000 mg daily methylprednisolone equivalent) as well as initiation of corticosteroids within the first 24 hours were associated with improved MACE-free survival in 90 days with early initiation appearing to have better outcomes regardless of the dose [10]. In our case presentation, both the dose and time to initiation of corticosteroids may have influenced the patient’s persistent CHB. Although the exact mechanisms are unclear, there may be an underlying dose-response mechanism that may be delineated in future studies.

While our patient was initiated on methylprednisolone 1000 mg IV daily, his first dose was administered roughly 48 hours into admission. His presentation was insidious with his initial rhythm of sinus bradycardia (estimated to account for 4.6% of adverse arrhythmic events) [5] in the backdrop of baseline conduction disease and no evidence of structural disease on TTE. He was also almost 90 days into durvalumab immunotherapy. Among 1141 cases of ICI-associated arrhythmia, the large majority of cases began within the first 30 days at 48.9% while only 11.0% of cases began at 61-90 days [5]. All of these were likely contributors to a delayed diagnosis. It was only until his disease manifested as CHB that methylprednisolone was initiated. While troponinemia resolved, CHB persisted and was complicated by TdP necessitating the implantation of a dual-chamber pacemaker with a defibrillator.

While there is limited data on the role of PPM implantation in ICI-associated myocarditis complicated by CHB, their use may prove to be a significant treatment strategy. A 2022 retrospective pooled analysis included 21 cases of CHB secondary to ICI-associated myocarditis with an overall fatality rate of 52%. Of the 21 cases, 16 patients received PPM with an overall fatality rate of 38% which was statistically significant compared to the five patients that did not receive pacemakers with an overall fatality rate of 100% [11]. This suggests a potential indication for PPM implantation, although no specific guidelines currently exist. The development of clinical guidelines supported by future studies may help clarify which specific populations undergoing ICI therapy may benefit most from PPM implantation.

## Conclusions

We describe a case of durvalumab-induced myocarditis presenting atypically with sinus bradycardia progressing to CHB. While the patient was initially treated with methylprednisolone with the resolution of troponinemia, CHB persisted and was complicated by polymorphic ventricular tachycardia, necessitating the implantation of a dual-chamber PPM. There is limited data on the treatment of ICI-associated myocarditis with most treatments extrapolated from treatment of myocarditis of other etiologies and only retrospective data analyzing such treatments, but the use of corticosteroids as well as PPM for patients with CHB seems to be promising. Prompt administration of corticosteroids seems to play a crucial role in patient outcomes, so it is important for clinicians to be aware of the adverse effects of ICIs, with vigilance to keep myocarditis in the differential as ICIs become more common in clinical practice.
